# CXCR4 induces podocyte injury and proteinuria by activating β-catenin signaling

**DOI:** 10.7150/thno.65948

**Published:** 2022-01-01

**Authors:** Hongyan Mo, Qian Ren, Dongyan Song, Bo Xu, Dong Zhou, Xue Hong, Fan Fan Hou, Lili Zhou, Youhua Liu

**Affiliations:** 1 State Key Laboratory of Organ Failure Research, National Clinical Research Center of Kidney Disease, Division of Nephrology, Nanfang Hospital, Southern Medical University, Guangzhou, China.; 2 Department of Pathology, University of Pittsburgh School of Medicine, Pittsburgh, Pennsylvania, USA.; 3 Division of Nephrology, The First Affiliated Hospital, Hengyang Medical School, University of South China, Hengyang, Hunan, China.; 4 Division of Nephrology, Department of Medicine, University of Connecticut School of Medicine, Farmington, Connecticut, USA.

**Keywords:** Podocyte, CXCR4, β-catenin, β-arrestin-1, proteinuria, glomerulosclerosis

## Abstract

**Background:** C-X-C chemokine receptor type 4 (CXCR4) plays a crucial role in mediating podocyte dysfunction, proteinuria and glomerulosclerosis. However, the underlying mechanism remains poorly understood. Here we studied the role of β-catenin in mediating CXCR4-triggered podocyte injury.

**Methods:** Mouse models of proteinuric kidney diseases were used to assess CXCR4 and β-catenin expression. We utilized cultured podocytes and glomeruli to delineate the signal pathways involved. Conditional knockout mice with podocyte-specific deletion of CXCR4 were generated and used to corroborate a role of CXCR4/β-catenin in podocyte injury and proteinuria.

**Results:** Both CXCR4 and β-catenin were induced and colocalized in the glomerular podocytes in several models of proteinuric kidney diseases. Activation of CXCR4 by its ligand SDF-1α stimulated β-catenin activation but did not affect the expression of Wnt ligands *in vitro*. Blockade of β-catenin signaling by ICG-001 preserved podocyte signature proteins and inhibited Snail1 and MMP-7 expression *in vitro* and *ex vivo*. Mechanistically, activation of CXCR4 by SDF-1α caused the formation of CXCR4/β-arrestin-1/Src signalosome in podocytes, which led to sequential phosphorylation of Src, EGFR, ERK1/2 and GSK-3β and ultimately β-catenin stabilization and activation. Silencing β-arrestin-1 abolished this cascade of events and inhibited β-catenin in response to CXCR4 stimulation. Podocyte-specific knockout of CXCR4 in mice abolished β-catenin activation, preserved podocyte integrity, reduced proteinuria and ameliorated glomerulosclerosis after Adriamycin injury.

**Conclusion:** These results suggest that CXCR4 promotes podocyte dysfunction and proteinuria by assembling CXCR4/β-arrestin-1/Src signalosome, which triggers a cascade of signal events leading to β-catenin activation.

## Introduction

Podocyte injury and proteinuria are the major pathological features of glomerular disease, which accounts for the vast majority of chronic kidney disease (CKD) leading to end-stage renal failure. As a key component of the glomerular filtration apparatus, the integrity of podocytes and their foot processes and slit diaphragm is essential for preventing proteinuria 1,2. Podocyte injury plays a crucial role in the pathogenesis of a wide variety of glomerular diseases ranging from focal segmental glomerulosclerosis (FSGS) to diabetic nephropathy (DN) 3,4. Studies have shown that podocyte damage and/or depletion often take place in the early stage of glomerular lesions when endothelial and mesangial cells remain relatively intact 5. Therefore, identification of the key mediators that regulate podocyte damage would be of great significance for developing interventional strategies to slow, halt or even reverse the progression of the majority of CKD.

Although many cues can cause podocyte injury, increasing evidence demonstrates that oxidative stress is an important common culprit of podocyte damage and proteinuria 6,7. Oxidative stress is often considered as the convergent pathway of many diverse triggers of podocyte damage 6-8. We have previously shown that C-X-C chemokine receptor type 4 (CXCR4), a seven transmembrane G-protein-coupled receptor (GPCR) specific for stromal-derived factor-1α (SDF-1α), also known as CXCL12, plays a key role in mediating oxidative stress-induced podocyte damage, proteinuria, and glomerulosclerotic lesions 8. CXCR4 is upregulated specifically in glomerular podocytes in numerous models of proteinuric CKD. Furthermore, inhibition of CXCR4 alleviates podocyte injury, proteinuria and glomerulosclerosis. Along this line, CXCR4 could be a new therapeutic target for clinical management of proteinuric CKD. However, how exactly CXCR4 activation causes podocyte damage remains to be elucidated.

Wnt/β-catenin is evolutionarily conserved signal pathway that plays an important role in regulating embryo development, tissue homeostasis, stem cell self-renewal, and tumorigenesis 9-11. In recent years, increasing numbers of studies have shown that Wnt/β-catenin is activated in a wide variety of CKD models induced by high glucose, Adriamycin (ADR), 5/6 nephrectomy (5/6NX), chronic angiotensin II infusion or protein overload 12-18. More importantly, podocyte-specific knockout of β-catenin prevents podocyte injury, ameliorates proteinuria and glomerulosclerotic lesions in ADR nephropathy 12,14, suggesting a causative role of β-catenin activation in mediating podocyte dysfunction. It is well known that β-catenin activation is primarily controlled by Wnt ligands. However, β-catenin can also be regulated by many other extracellular cues beyond Wnts, such as MMP-7 through inducing degradation of E-cadherin 19, as well as CXCR4 signaling in tumor cells 20,21. However, whether there is a link between CXCR4 and Wnt/β-catenin in podocyte injury and proteinuria remains to be determined.

In this study, we report that CXCR4 in glomerular podocytes activates β-catenin signaling via forming a CXCR4/β-arrestin-1/Src signalosome. Podocyte-specific deletion of CXCR4 inhibits β-catenin activation, reduces proteinuria, ameliorates podocyte injury and glomerulosclerosis. Our results suggest that targeting CXCR4/β-arrestin-1/β-catenin may be a new strategy for the treatment of proteinuric CKD.

## Materials and methods

### Mice and genotyping

Homozygous CXCR4-floxed mice in C57BL/6J background were obtained from the Jackson Laboratories (Stock No. 008767; Bar Harbor, ME). Transgenic mice expressing Cre recombinase under the control of human podocin (*NPHS2*) promoter (Podo-Cre) was also obtained from Jackson Laboratories (Stock No. 008205). By mating CXCR4-floxed mice with Podo-Cre transgenic mice, conditional knockout mice (Podo-CXCR4-/-) in which *CXCR4* gene was specifically disrupted in podocyte (genotype: CXCR4^fl/fl^; Cre^+/-^) were created. These mice were crossbred with homozygous CXCR4-floxed mice to generate offspring with 50% Podo-CXCR4-/- mice and 50% control mice (genotype: CXCR4^fl/fl^; Cre^-/-^) within the same litters. A routine PCR protocol was used for genotyping of tail DNA samples with the primer pairs as described in Table S1. All animals were born normally at the expected Mendelian frequency; and they were normal in size and did not display any gross physical or behavioral abnormalities. Animal experiments were approved by the Institutional Animal Care and Use Committee at the University of Pittsburgh and Animal Ethics Committee at the Nanfang Hospital.

### Animal models

Male BALB/c mice weighing 22~24g were obtained from the Southern Medical University Animal Center (Guangzhou, China). For ADR nephropathy model, BALB/c mice were administered by a single intravenous injection of ADR (doxorubicin hydrochloride; Sigma-Aldrich, St. Louis, MO) at 10 mg/kg body weight. Groups of mice (n=6) were euthanized at 1 and 3 weeks after ADR injection, and kidney tissues collected for various analyses. Because mice with C57BL/6J background are resistant to ADR nephropathy, podo-CXCR4-/- and CXCR4-floxed (control) mice were administered with two intravenous injections of ADR at 15 mg/kg body weight, one week apart. Groups of mice (n=7) were euthanized at 2 weeks after ADR injection, and kidney tissues collected for various analyses. In addition, mouse models of proteinuric kidney diseases including angiotensin II infusion (520 ng/min/kg for 4 weeks), db/db mice (5 months) and remnant kidney model after 5/6 nephrectomy (5/6NX) (8 weeks) were used, as described previously 22,23.

### SDF-1α ELISA

The enzyme-linked immunosorbent assay (ELISA) kit for mouse SDF-1α was purchased from the Cusabio Company (CSB-EQ027494MO; Wuhan, China). Serum SDF-1α level was measured according to the assay procedures specified by the manufacturer. The enzymatic reaction products were quantified in an automated microplate reader. Serum SDF-1α levels were expressed as nanogram per milliliter.

### Cell culture and treatment

The conditionally immortalized mouse podocyte cell line (MPC5) was provided by Peter Mundel (Massachusetts General Hospital, Boston, MA). To propagate podocytes, cells were cultured at 33 °C in RPMI-1640 medium supplemented with 10% fetal bovine serum (FBS) and 10 units/ml mouse recombinant IFN-γ (R&D Systems, Minneapolis, MN) to enhance the expression of a thermosensitive T antigen. To induce differentiation, podocytes were grown under nonpermissive conditions at 37° C in the absence of IFN-γ. MPC5 cells were treated with SDF-1α (SRP-4388; Sigma) with different doses for various periods of time as indicated. For some experiments, MPC5 cells were pretreated with CXCR4 inhibitor AMD3100 (A5602; Sigma) at 5 µg/mL, ICG-001 (Chembest, Shanghai, China) at 5 µM and MEK1/2 inhibitor U0126 (9903; Cell Signaling Technology) at 10 µM. For some studies, podocytes were transiently transfected with ON-TARGET plus SMART pool siRNA (400 pM) specific for mouse β-arrestin-1 or control nontarget siRNA using Lipofectamine 2000 reagent according to the manufacturer's instruction (Invitrogen). Forty-eight hours after transfection, podocytes were exposed to 100 ng/mL SDF-1α for 6 h and harvested for various analyses.

### Glomerular culture

Glomeruli were isolated by differential sieving technique from male Sprague Dawley rats (Harlan Sprague Dawley), as previously reported 8. Briefly, kidneys were excised and pressed with a spatula through stainless steel screens through differential sieves (60, 100, and 200 meshes) and collected for cultivation. The purity of glomeruli was about 95% using this approach 8. Isolated glomeruli were cultured in the noncoated 24-well plates with SDF-1α in the absence or presence of AMD3100 or ICG-001, respectively.

### Urinary albumin, creatinine assay

Urinary albumin was measured by using a mouse Albumin ELISA Quantitation kit, according to the manufacturer's protocol (Bethyl Laboratories, Inc., Montgomery, TX). Urinary and serum creatinine levels were determined by use of a QuantiChrom creatinine assay kit (DICT-500; Bioassay Systems, Hayward, CA), according to the manufacturer's protocols. Urinary albumin was standardized to urine creatinine and expressed as mg/mg Ucr.

### Histology and immunohistochemical staining

Paraffin-embedded mouse kidney sections (3 µm thickness) were prepared and stained with periodic acid-Schiff (PAS) and Masson's trichrome staining (MTS) reagents. Immunohistochemical staining was performed using established protocols, as described previously 8. The antibodies used were described in Table S2.

### Immunofluorescence staining and confocal microscopy

Human kidney specimens were collected from diagnostic kidney biopsies performed at Nanfang Hospital, Southern Medical University. Non-tumor renal tissues from patients who had renal cell carcinoma and underwent nephrectomy were used as a normal control. Human kidney cryosections (3 μm thickness) were prepared by a routine method. The studies involving human samples were approved by the Medical Ethics Committee at the Nanfang Hospital, Southern Medical University.

Kidney cryosections or cells cultured on coverslips were fixed with 4% paraformaldehyde for 15 min at room temperature. After blocking with 10% donkey serum for 1 h, the slides were immunostained with various primary antibodies as described in Table S2. Sections from diseased kidneys were also stained with isotype control antibodies and no specific staining occurred.

### Western blot analysis

Protein expression was analyzed by Western blot analysis, as described previously 8. The detail information of the antibodies used were described in Table S2. Relative protein levels of Western blots were quantified with ImageJ software and reported after normalizing to the loading control.

### Co-immunoprecipitation

The interaction of CXCR4 or Src with β-arrestin-1 in mouse podocytes treated with SDF-1α for 5 min was determined by co-IP as previously described 24. Cell lysates were immunoprecipitated overnight at 4 °C with anti-CXCR4 or anti-β-arrestin-1 antibodies and protein A/G plus agarose (sc-2003; Santa Cruz Biotechnology), respectively. The precipitated complexes were washed with lysis buffer and boiled for 5 min in SDS sample buffer followed by immunoblotting with anti-CXCR4, and anti-Src anti-β-arrestin-1 antibodies, respectively. The antibodies used were described in Table S2.

### Real-time RT-PCR

Total RNA isolation and real-time quantitative qRT-PCR were carried out by procedures described previously 7. Briefly, first-strand cDNA synthesis was carried out by using a Reverse Transcription System Kit according to the instructions of the manufacturer (Promega). Real-time RT-PCR was performed on an ABI PRISM 7000 Sequence Detection System (Applied Biosystems, Foster City, CA). The PCR reaction mixture in a 25-µL volume contained 12.5 µL 2X SYBR Green PCR Master Mix (Applied Biosystems), 5 µL diluted RT product (1:10), and 0.5 µM sense and antisense primer sets. PCR reaction was run by using standard conditions. After sequential incubations at 50 °C for 2 min and 95 °C for 10 min, the amplification protocol consisted of 50 cycles of denaturing at 95 °C for 15 s and annealing and extension at 60 °C for 60 s. The mRNA levels of various genes were calculated after normalizing with β-actin. Primer sequences used in this study were listed in Table S1.

### Statistical analyses

All data examined were expressed as mean ± standard error of the mean. Statistical analysis of the data was carried out using SPSS 19.0, (SPSS, Inc., Chicago, IL). Comparison between groups was made using one-way analysis of variance (ANOVA) followed by Student-Newman-Keuls test or Dunnett's T3 procedure. *P* < 0.05 was considered significant.

## Results

### Podocyte injury is associated with CXCR4 and β-catenin induction in proteinuric CKD

**Figure 1:**
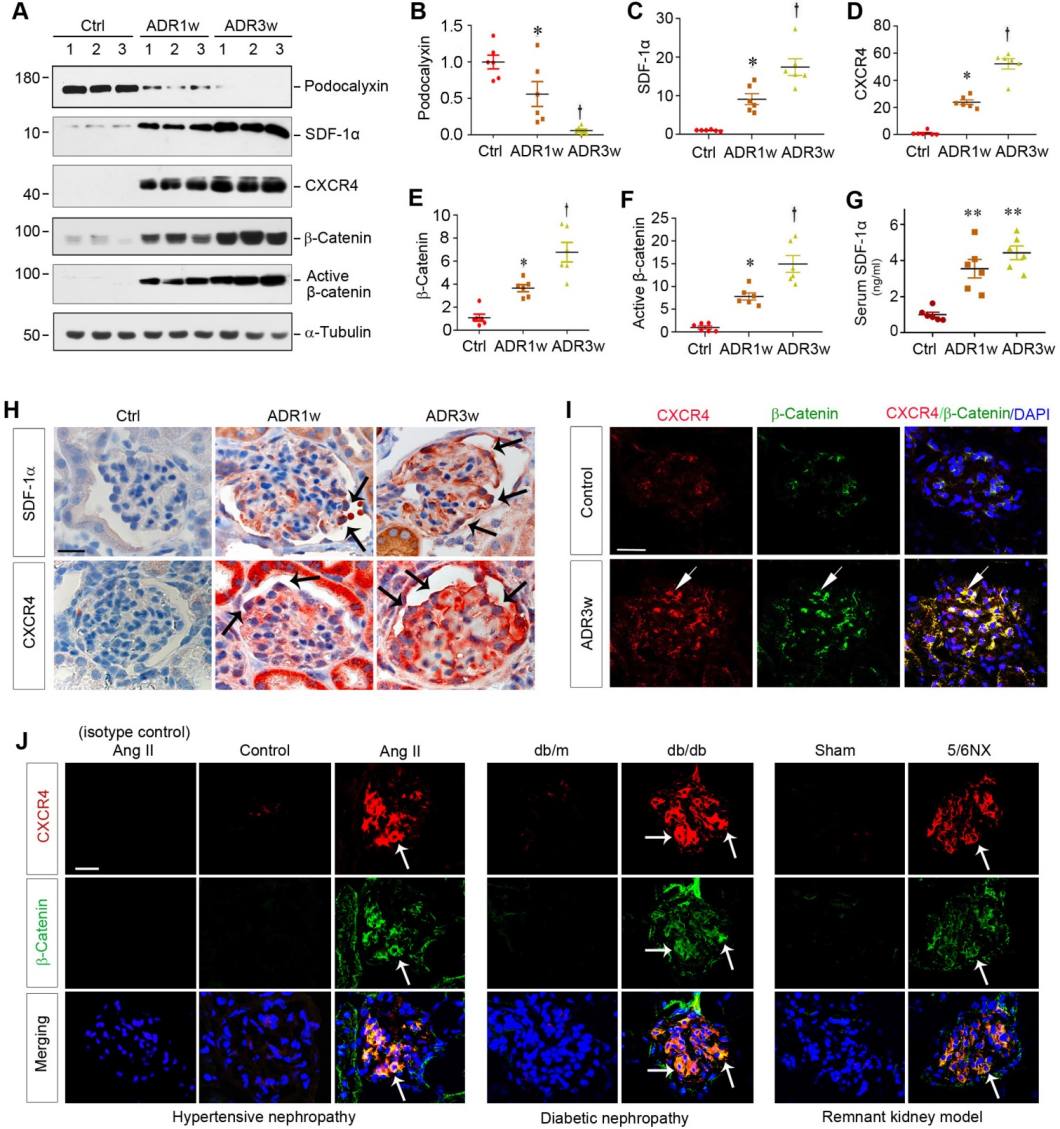
** Podocyte injury is associated with CXCR4 induction and β-catenin activation in mouse models of proteinuric kidney diseases.** (**A-F**) Western blot analyses of renal expressions of podocalyxin, SDF-1α, CXCR4, total and active β-catenin in different groups. Numbers (1, 2 and 3) represent different animals in a given group. Graphical representations of podocalyxin (**B**), SDF-1α (**C**), CXCR4 (**D**), total β-catenin (**E**) and active β-catenin (**F**) expressions in different groups as indicated. ^*^P < 0.05 versus normal controls (n = 6), ^†^*P* < 0.05 versus ADR 1 week (n = 6). (**G**) Serum levels of SDF-1α assessed by ELISA. The circulating levels of SDF-1α was assessed by a specific ELISA in different groups as indicated. ^**^P < 0.01 versus normal controls (n = 6). (**H**) Representative micrographs show the expression and localization of SDF-1α and CXCR4 in mouse glomeruli after ADR injection. SDF-1α and CXCR4 expression in different groups was shown as indicated. Arrow indicates positive staining. Scale bar, 20 µm. (**I**) Colocalization of CXCR4 and β-catenin in the glomeruli at 3 weeks after ADR injection. Kidney sections were immunostained for CXCR4 and β-catenin. Colocalizations of CXCR4 and β-catenin in glomerular podocytes are indicated by arrows. Scale bar, 20 µm. (**J**) Colocalization of CXCR4 and β-catenin in the glomeruli of mouse models of proteinuric CKD. Kidney cryosections from different mouse models of proteinuric CKD as indicated were immunostained for CXCR4 (red) and β-catenin (green). Colocalizations of CXCR4 and β-catenin in glomeruli are indicated by arrows. Scale bar, 20 µm.

ADR nephropathy, a model of human FSGS, is characterized by podocyte injury, proteinuria and glomerulosclerosis 8,25. As shown in Figure 1A-B, ADR caused glomerular damage and inhibited podocyte-specific podocalyxin expression in a time-dependent manner. Interestingly, loss of podocalyxin was closely associated with SDF-1α and CXCR4 induction and β-catenin activation at 0, 1, and 3 weeks after ADR injection, as shown by Western blot analyses of whole kidney lysates (Figure 1A-F). Serum levels of SDF-1α protein were also elevated in mice at different time points after ADR injection (Figure 1G).

Immunohistochemical staining confirmed that both SDF-1α and CXCR4 were upregulated in glomerular podocytes and, to a less extent, tubular epithelial cells after ADR injection (Figure 1H). To further investigate the potential relationship between CXCR4 and β-catenin, we utilized double immunofluorescence staining for CXCR4 and β-catenin. As shown in Figure 1I, both CXCR4 and β-catenin were colocalized in the glomeruli at 3 weeks after ADR injection.

To extend this finding, we further examined the expression of CXCR4 and β-catenin in several common models of proteinuric CKD with different etiologies. As shown in Figure 1J, both CXCR4 (red) and β-catenin (green) were induced and colocalized in the glomeruli of mice in hypertensive nephropathy induced by chronic infusion of angiotensin II (Ang II), diabetic nephropathy in genetic db/db mice, and remnant kidney model induced by 5/6NX. Furthermore, CXCR4 was also induced and colocalized with β-catenin in the glomeruli of human kidney biopsies from patients with proteinuric CKD including DN, FSGS and membranous nephropathy (MN) (Figure S1). These results suggest a potential connection between CXCR4 induction and β-catenin activation in the glomerular podocytes in a variety of proteinuric CKD.

### Activation of CXCR4 activates β-catenin by a Wnt-independent mechanism

**Figure 2:**
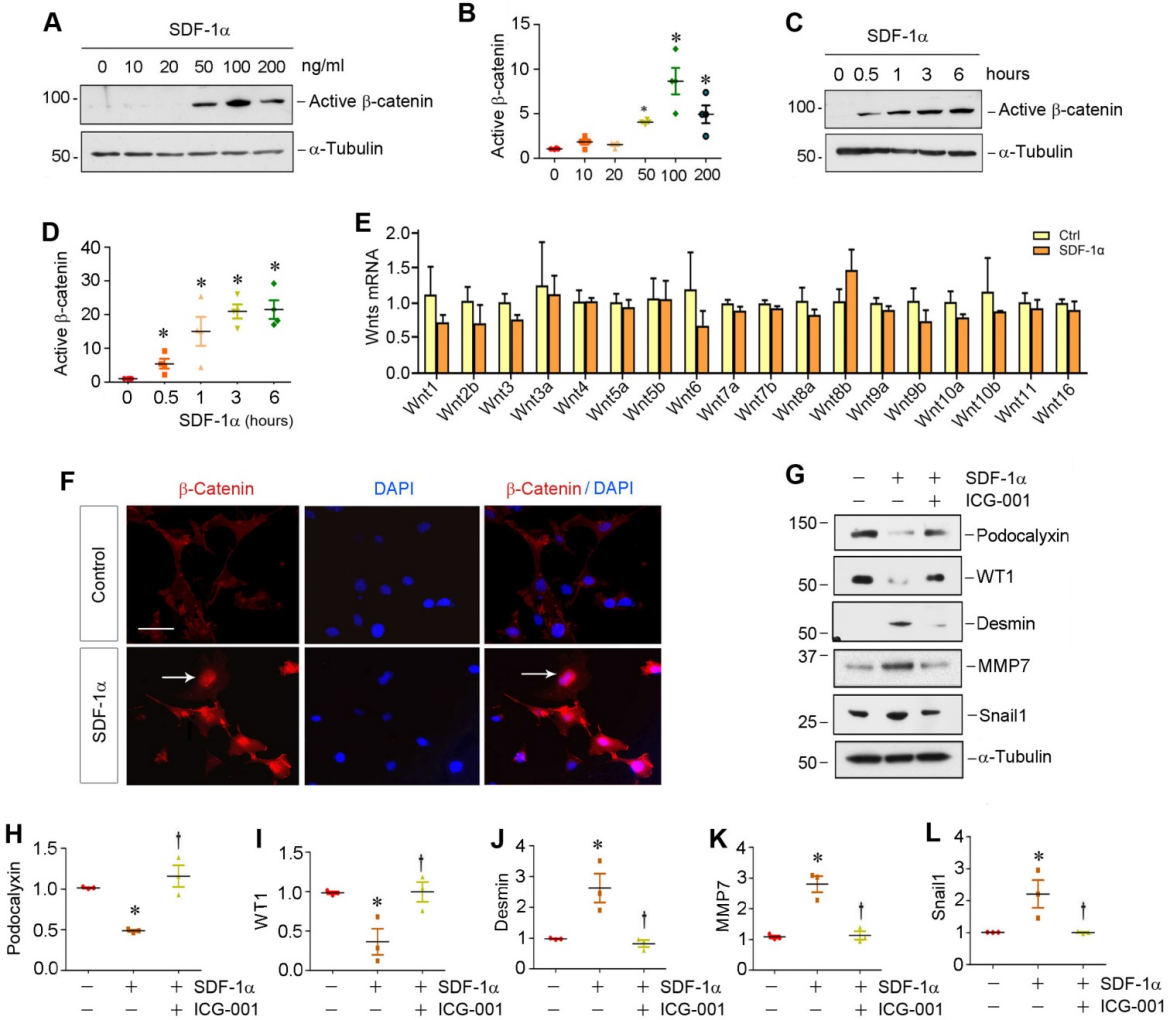
** SDF-1α/CXCR4 signaling activates β-catenin without affecting Wnt expression in podocytes *in vitro*.** (**A**) Western blotting shows that SDF-1α induced β-catenin activation in a dose-dependent manner. Mouse podocyte cell line (MPC-5) was treated with SDF-1α (0~200 ng/mL) for 6 h. Whole cell lysates were immunoblotted with specific antibodies against active β-catenin or α-tubulin, respectively. (**B**) Graphical representation of the relative abundances of active β-catenin in different groups as indicated. ^*^P < 0.05 versus controls (n = 3). (**C**, **D**) Western blotting shows that SDF-1α induced active-β-catenin expression in a time-dependent manner. MPC5 cells were treated with SDF-1α at dose of 100 ng/mL for various periods of time as indicated. Representative Westtern blot (**C**) and quantitative data (**D**) are presented. ^*^P < 0.05 versus controls (n = 3). (**E**) Quantitative real-time PCR (qPCR) analyses show mRNA expression of Wnt ligands in cultured podocytes after treated with SDF-1α (100 ng/mL) for 0.5 h (n=3). (**F**) Immunofluorescence micrographs show β-catenin staining in cultured podocytes after treated with SDF-1α. β-Catenin was increased by SDF-1α (100 ng/mL) and trans-localized to the nucleus. Arrow indicates positive staining for β-catenin. Scale bar, 20 µm. (**G**-**L**) Representative Western blotting (**G**) and quantitative analyses of podocalyxin (**H**), WT1 (**I**), desmin (**J**), MMP-7 (**K**) and Snail1 (**L**) in different groups as indicated. ^*^P < 0.05 versus controls (n = 3); ^†^*P* < 0.05 versus SDF-1α alone (n = 3).

To investigate the relationship between β-catenin and CXCR4, we examined the regulation of β-catenin after CXCR4 activation in cultured podocytes *in vitro*. To this end, mouse podocytes (MPC5) were treated with various doses of SDF-1α, the ligand of CXCR4. As illustrated in Figure 2A-B, SDF-1α induced β-catenin activation in cultured podocytes, which peaked when SDF-1α was at 100 ng/mL, as demonstrated by Western blotting using specific antibody against active β-catenin. Time-course studies revealed that β-catenin activation occurred rapidly, starting as early as 0.5 h, after SDF-1α incubation (Figure 2C-D).

We then investigated the expression of the Wnt ligands in mouse podocytes. As shown in Figure 2E, a comprehensive survey was performed to assess the expression of various Wnt ligands in MPC5 cells after treatment with 100 ng/mL SDF-1α for 0.5 h by quantitative real-time RT-PCR (qRT-PCR). We found no significant change in the mRNA expression of all Wnts tested, suggesting that SDF-1α/CXCR4 signaling activates β-catenin by a mechanism independent of Wnt induction. Similar results were obtained when MPC5 cells were incubated with SDF-1α for 24 h (Figure S2).

### Activation β-catenin mediates SDF-1α/CXCR4-triggered podocyte injury

We further investigated β-catenin localization and its functional consequence after SDF-1α stimulation. As shown in Figure 2F, β-catenin predominantly localized in the nuclei of podocytes after SDF-1α treatment, indicating its nuclear translocation. As earlier studies indicate that activation of β-catenin impairs podocyte function and causes albuminuria 12,18,24, we further examined the regulation of podocyte-specific markers such as podocalyxin and Wilms tumor 1 (WT1), as well as podocyte injury marker desmin and β-catenin downstream targets matrix metalloproteinase-7 (MMP-7) and Snail1. As shown in Figure 2G-L, incubation of podocytes with SDF-1α repressed podocalyxin and WT1 expression and induced desmin, MMP-7 and Snail1. However, preincubation with ICG-001, an inhibitor of β-catenin-mediated transcription through disrupting β-catenin/CBP interaction 26,27, significantly preserved the expression of podocalyxin and WT1. ICG-001 also abolished the induction of desmin, MMP-7 and Snail1 triggered by SDF-1α (Figure 2G, J-L). Similarly, blocking β-catenin signaling by ICG-001 resulted in restoration of zona occludens 1 (ZO-1) suppressed by SDF-1α in podocytes (Figure S3). These results suggest that β-catenin activation mediates SDF-1α/CXCR4-triggered podocyte injury.

### SDF-1α induces β-catenin activation by forming CXCR4/β-arrestin 1/Src signalosome

We sought to delineate how CXCR4 activation by SDF-1α leads to an increased β-catenin signaling in podocytes. Earlier studies suggest that β-arrestin-1, a scaffold protein, plays a crucial role in mediating the signal transduction of G protein-coupled receptor 28-30. To study a potential involvement of β-arrestin-1 in proteinuric kidney diseases, we examined its expression in the nephropathies induced by ADR or chronic infusion of angiotensin II, which are associated with increased levels of oxidative stress and CXCR4 8,31,32. As shown in Figure S4, renal expression of β-arrestin-1 mRNA was induced in both models, suggesting a possible role of β-arrestin-1 in mediating CXCR4-triggered podocyte injury.

**Figure 3:**
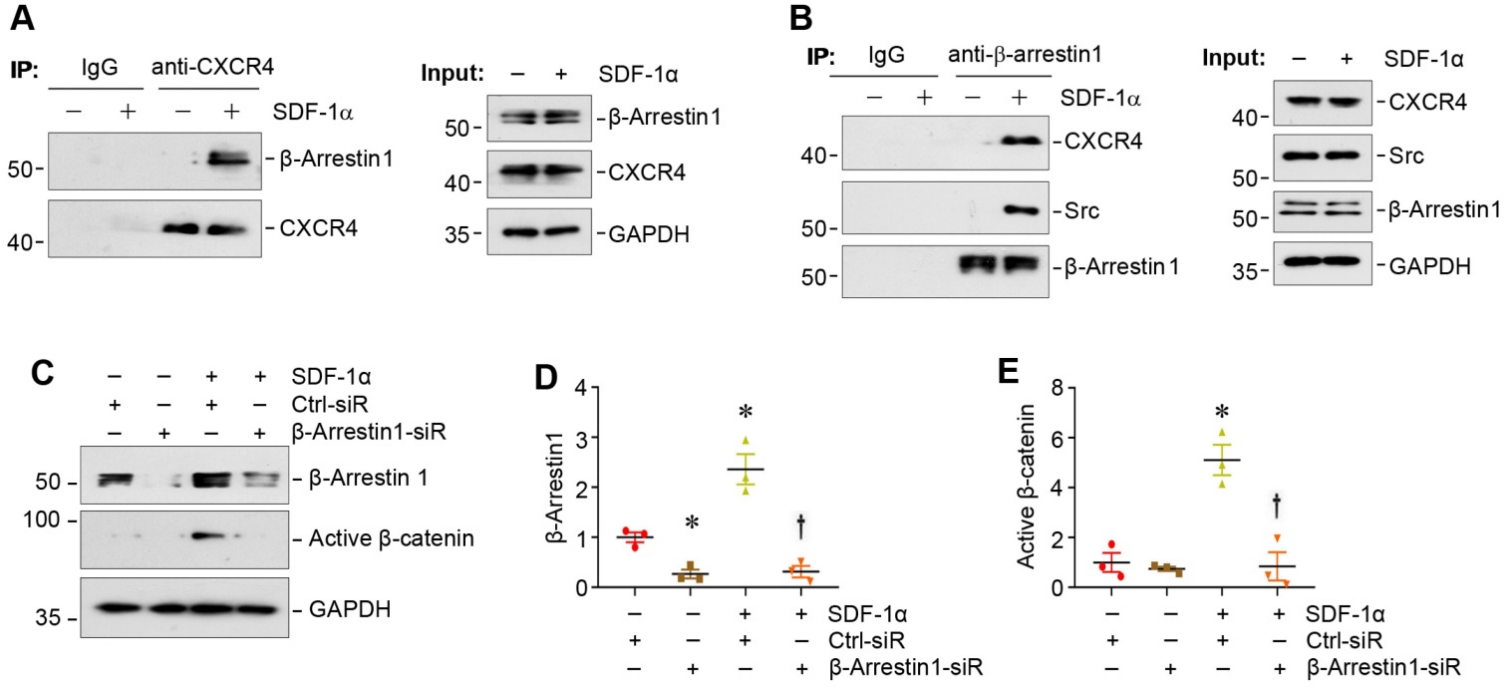
** SDF-1α induces β-catenin activation by forming the signaling complex of CXCR4/β-arrestin-1/Src in podocytes.** (**A**) Immunoprecipitation (IP) revealed the association of CXCR4 with β-arrestin-1 in podocytes. MPC5 cells were treated with SDF-1α for 5 min and cell lysates were immunoprecipitated with anti-CXCR4 antibody, followed by immunoblotting (IB) with anti-β-arrestin-1 and anti-CXCR4 antibodies. The expression of β-arrestin-1, CXCR4 and GAPDH in cell lysates was used as input. (**B**) IP demonstrated that SDF-1α induced the complex formation of CXCR4/β-arrestin-1/Src in podocytes. Cell lysates were immunoprecipitated with anti-β-arrestin-1 antibody, followed by immunoblotting with antibodies against CXCR4, Src or β-arrestin-1, respectively. The levels of CXCR4, Src, β-arrestin-1 and GAPDH in cell lysates were shown by Western blotting (Input). (**C**-**E**) Western blotting shows that knockdown of β-arrestin-1 abolished β-catenin activation induced by SDF-1α in podocytes. Representative Western blot (**C**) and quantitative data of β-arrestin-1 (**D**) and active β-catenin (**E**) are presented. ^*^P < 0.05 versus controls (n = 3), ^†^*P* < 0.05 versus SDF-1α (n = 3).

We next investigated the potential role of β-arrestin-1 in SDF-1α/CXCR4-induced β-catenin activation. Co-immunoprecipitation (co-IP) studies showed that SDF-1α promoted the association between CXCR4 and β-arrestin-1 (Figure 3A), which was evident at 5 min after SDF-1α treatment. As shown in Figure 3B, SDF-1α promoted the formation of the signalosome consisting of CXCR4/β-arrestin-1/Src kinase, as illustrated by the immunocomplex formation among CXCR4, Src and β-arrestin-1 in podocytes at 5 min after SDF-1α treatment. Of note, the abundance of CXCR4 was not altered in mouse podocytes after treatment with SDF-1α (Figure 3B). To determine whether the formation of CXCR4/β-arrestin-1/Src is indispensable for SDF-1α-induced β-catenin activation, we assessed β-catenin activation in the podocytes with β-arrestin-1 depletion. As presented in Figure 3C-E, knockdown of β-arrestin-1 by small interfering RNA (siRNA) resulted in significant reduction of active β-catenin in podocytes after treatment with SDF-1α.

### CXCR4/β-arrestin-1/Src signalosome promotes EGFR activation and ERK/GSK3β phosphorylation

**Figure 4:**
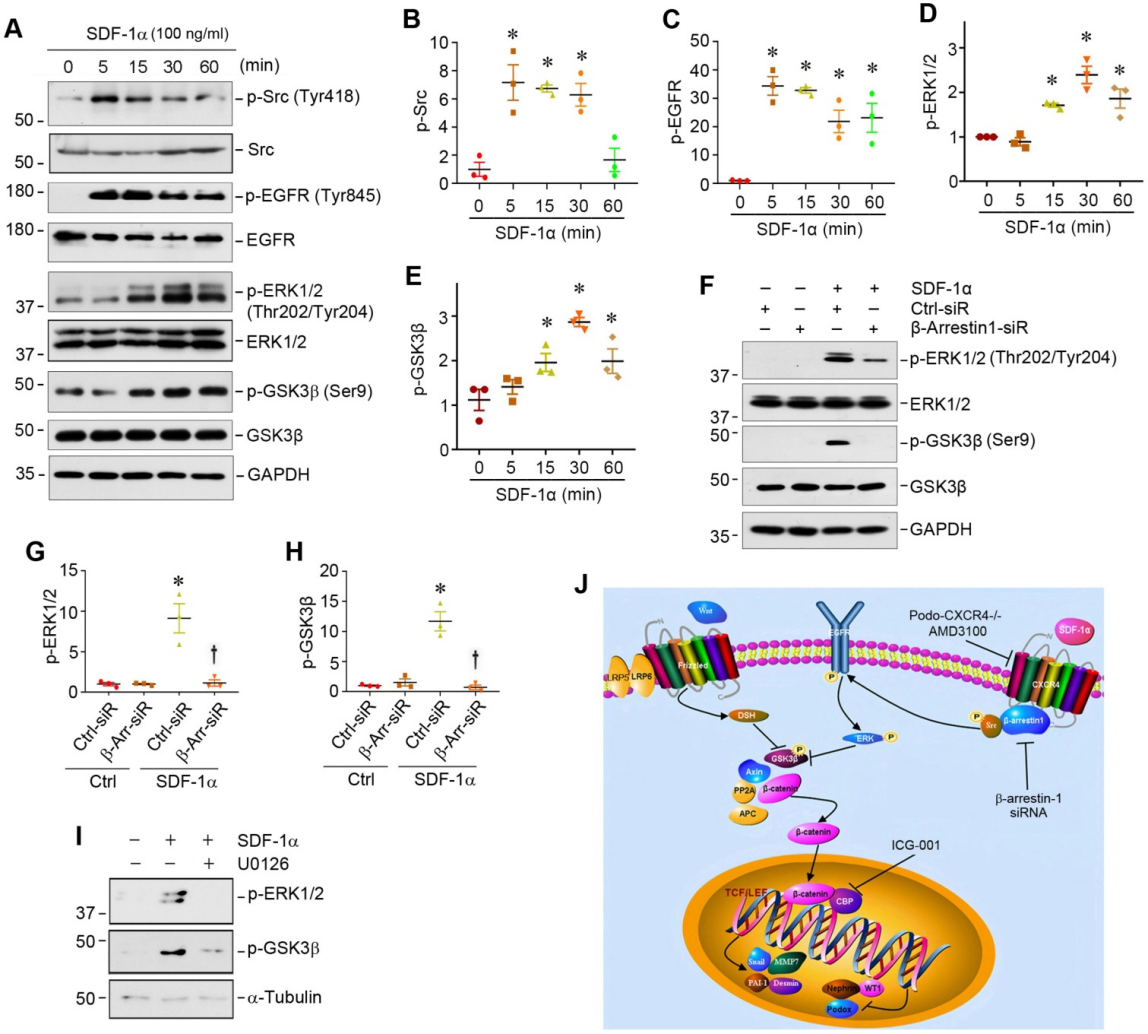
** SDF-1α/CXCR4/β-arrestin-1/Src signalosome promotes EGFR activation and triggers its downstream ERK1/2/GSK3β phosphorylation.** (**A-E**) Western blotting shows that SDF-1α induced Src, EGFR, ERK1/2, GSK3β phosphorylation in cultured podocytes. MPC5 cells were treated with SDF-1α (100 ng/ml) for various periods as indicated. Representative Western blot (**A**) and quantitative data on p-Src (**B**), p-EGFR (**C**), p-ERK1/2 (**D**) and p-GSK3β (**E**) are shown. ^*^P < 0.05 versus controls (n = 3). (**F-H**) Western blotting and graphical representation show that knockdown of β-arrestin-1 reduced SDF-1α induced ERK1/2 and GSK3β phosphorylation in cultured podocytes. MPC5 cells were transfected with control or β-arrestin-1-specific siRNA. Representative Western blot (**F**) and quantitative data on the expression of p-ERK1/2 (**G**) and p-GSK3β (**H**) are shown. ^*^P < 0.05 versus controls (n = 3), ^†^*P* < 0.05 versus SDF-1α (n = 3). (**I**) Western blotting shows that blockade of ERK1/2 phosphorylation by U0126 abolished SDF-1α induced GSK3β phosphorylation in cultured podocytes. (**J**) Diagram depicts the signal transduction pathway that mediates SDF-1α activation of β-catenin in podocytes. The formation of CXCR4/β-arrestin-1/Src signalosome promotes sequential phosphorylation of EGFR/ERK1/2/GSK3β, leading to β-catenin activation. Inhibition of CXCR4 by AMD3100, knockdown of β-arrestin-1 by siRNA or inhibition of β-catenin signaling by ICG-001 can block this signaling in podocytes.

We further studied the downstream signaling of CXCR4/β-arrestin-1/Src signalosome after SDF-1α stimulation in podocytes. As activation of Src leads to epidermal growth factor receptor (EGFR) transactivation 33, which could cause extracellular signal-regulated kinase-1/2 (ERK1/2) phosphorylation 34,35, we explored whether the CXCR4/β-arrestin-1/Src signalosome induces EGFR activation and ERK1/2 phosphorylation. As presented in Figure 4A, Western blot analysis revealed an increase in the phosphorylation of Src at Tyr418, EGFR at Tyr845 and ERK1/2 at Thr202/Tyr204 in the MPC5 cells treated with SDF-1α. SDF-1α also induced GSK3β phosphorylation at Ser9, leading to its inactivation. Time-course studies showed that the induction of p-Src and p-EGFR occurred as early as 5 min, while ERK1/2 and GSK-3β phosphorylation started at 15 min and reached the peak at 30 min after SDF-1α stimulation (Figure 4A-E). This cascade of sequential phosphorylation of EGFR/ERK1/2/GSK-3β was dependent on CXCR4/β-arrestin-1/Src signalosome, as knocking down of β-arrestin-1 by siRNA suppressed ERK1/2 and GSK-3β phosphorylation in podocytes (Figure 4F-H). Preincubation with U0126, a highly selective inhibitor of ERK upstream MEK1 and MEK2, abolished the SDF-1α-induced ERK1/2 and GSK-3β phosphorylation (Figure 4I), confirming that ERK1/2 is an upstream regulator of GSK-3β phosphorylation. Because GSK-3β is an important component of the destruction complex of β-catenin, phosphorylation leads to its inactivation and disables its ability to phosphorylate downstream β-catenin 36. Taken together, as shown in Figure 4J, activation of CXCR4 by SDF-1α stimulates β-catenin signaling through a complex mechanism involving β-arrestin-1, Src, EGFR, ERK1/2 and GSK-3β in podocytes.

### SDF-1α/CXCR4 induces podocyte injury through β-catenin activation in glomerular miniorgan culture

**Figure 5:**
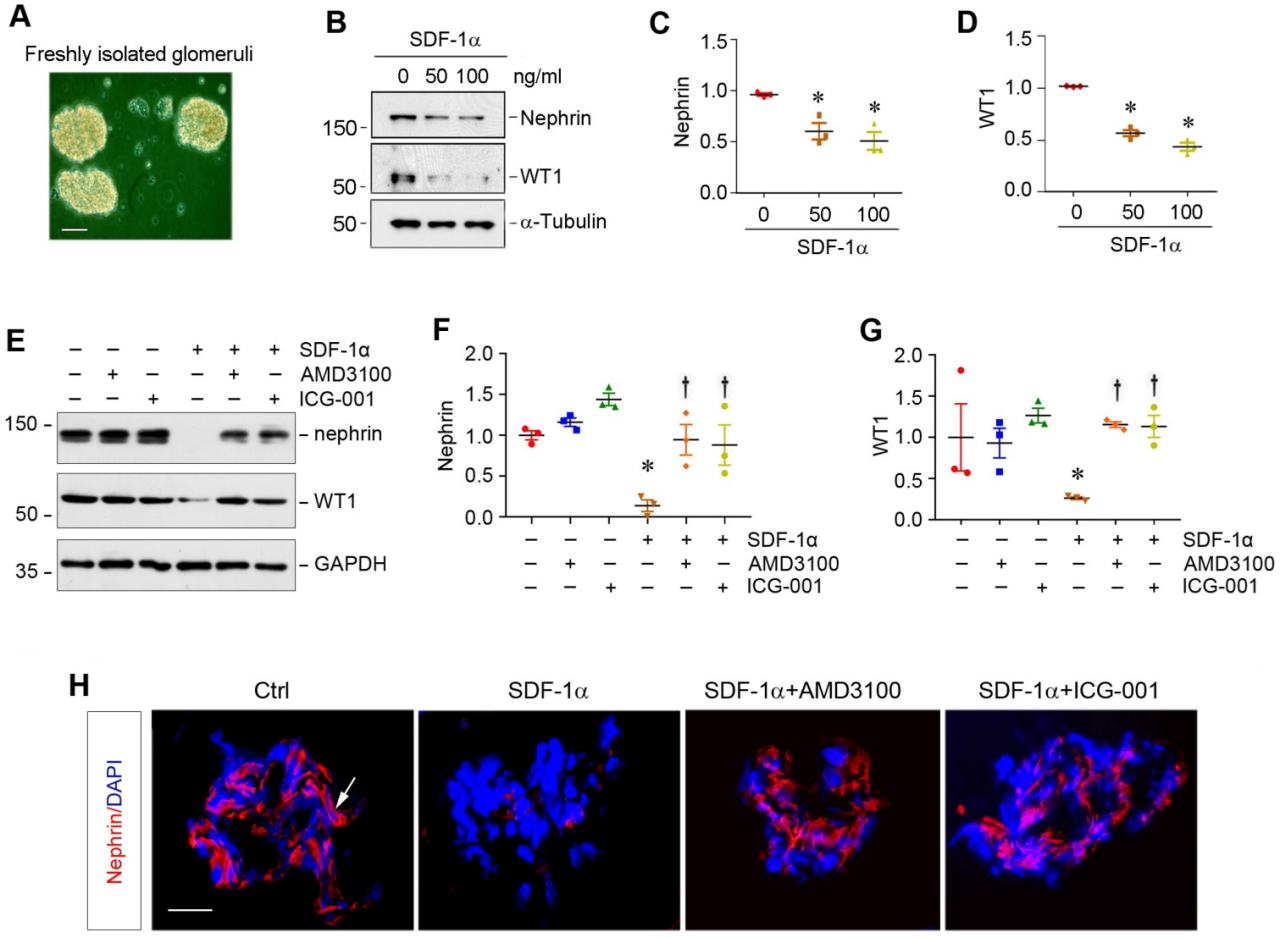
** SDF-1α/CXCR4 induces podocyte injury in glomerular mini-organ culture.** (**A**) Representative micrograph shows rat glomerular mini-organ culture *in vitro*. Glomeruli were isolated from rat kidneys and cultivated in suspension. Scale bar, 20 µm. (**B**-**D**) Western blotting shows that SDF-1α inhibited nephrin and WT1 expression in cultured glomeruli. Rat glomeruli were treated with SDF-1α (50 or 100 ng/mL) for 24 h. Quantitative data show nephrin (**C**) and WT1 (**D**) expression in different groups as indicated. ^*^P < 0.05 versus controls (n = 3). (**E**-**G**) Western blotting shows that blockade of CXCR4 by AMD3100 or β-catenin signaling by ICG-001 restored nephrin and WT1 expression in cultured glomeruli. Rat glomeruli were pretreated with AMD3100 (5 ng/mL) or ICG-001 (5 µM) for 1 h and then treated with SDF-1α (100 ng/mL) for 24 h. Quantitative data on nephrin (**F**) and WT1 (**G**) are presented. ^*^P < 0.05 versus controls (n = 3), ^†^*P* < 0.05 versus SDF-1α (n = 3). (**H**) Immunofluorescence staining shows that that blockade of CXCR4 by AMD3100 or β-catenin signaling by ICG-001 restored nephrin repressed by SDF-1α in cultured glomeruli. Arrow, positive staining. Scale bar, 20 µm.

To closely model podocytes *in vivo*, we used glomerular mini-organ culture, an *ex vivo* model system that largely preserves the sophisticated three dimensional architecture of podocytes in the kidney 24,37. As shown in Figure 5A, rat glomeruli were isolated by differential sieving technique and cultured in suspension, which retained relatively intact glomerular structure. Incubation with SDF-1α significantly inhibited nephrin and WT1 expression in cultured glomeruli (Figure 5B-D). However, blockade of either CXCR4 signaling by AMD3100 or β-catenin signaling by ICG-001 could largely restore nephrin and WT1 (Figure 5E-G). Similar results were obtained when nephrin was assessed by immunofluorescence staining (Figure 5H).

### CXCR4 is dispensable for podocyte physiology and function *in vivo*

**Figure 6:**
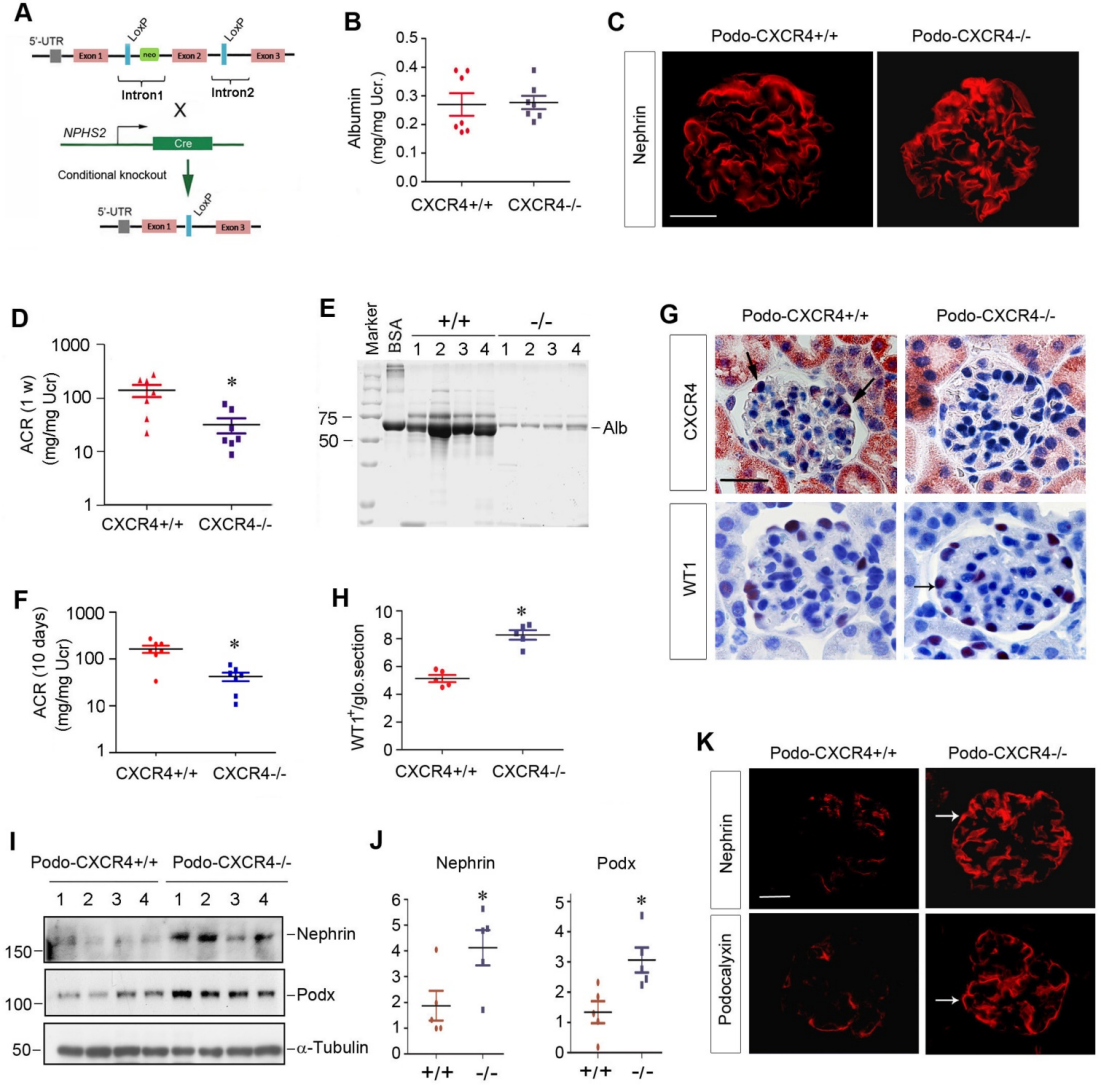
** Podocyte-specific ablation of CXCR4 preserves podocyte integrity and mitigates proteinuria after injury.** (**A**) Experimental design shows the strategy of crossbreeding of the CXCR4-floxed mice (CXCR4^fl/fl^) with Cre transgenic mice under the control of podocin promoter (podo-Cre). Pink boxes indicate the exons of CXCR4 gene. Blue boxes denote LoxP site. (**B**) Urinary albumin levels in mice in normal conditions. Urinary albumin is expressed as mg/mg of creatinine (n = 7). (**C**) Immunofluorescence micrographs show nephrin staining in the WT and KO mice in normal conditions. Scale bar, 25 µm. (**D**) Urinary albumin levels in podo-CXCR4+/+ and podo-CXCR4-/- mice at 1 week after ADR injection. Urinary albumin is expressed as mg/ mg of creatinine. ^*^P <0.05 versus podo-CXCR4+/+ (n = 7). (**E**) SDS-PAGE analysis shows the abundance and composition of urinary proteins in different groups of mice at 1 week after ADR injection. Urine samples after normalization to creatinine were analyzed on SDS- PAGE, with BSA (1 µg) loaded on the adjacent lane. The numbers (1-4) indicate each individual animal in a group. (**F**) Urinary albumin levels in mice at 10 days after ADR injection. Urinary albumin is expressed as mg/ mg of creatinine. ^*^P <0.05 versus podo-CXCR4+/+ (n = 7). (**G**) Representative micrographs show CXCR4 and WT1 staining in different groups at 2 weeks after ADR. Arrows indicate positive staining. Scale bar, 30 µm. (**H**) Quantitative analyses show the number of WT1^+^ cells in each glomerular section in different groups as indicated. ^*^P <0.05 (n = 5). (**I**, **J**) Western blot analyses show protein abundance of nephrin and podocalyxin in different groups. Representative Western blot (**I**) and quantitative data (**J**) are presented. ^*^P < 0.05 (n = 5). Podx, podocalyxin. (**K**) Representative micrographs show nephrin and podocalyxin staining in different groups as indicated. Arrows indicate positive staining. Scale bar, 20 µm.

We sought to determine the potential role of CXCR4 in podocyte physiology and pathology *in vivo*. To this end, we generated conditional knockout mice in which CXCR4 gene is specifically disrupted in podocytes. Homozygous CXCR4-floxed mice were mated with podo-Cre transgenic mice expressing Cre recombinase under the control of podocin promoter (Figure 6A). As shown in Figure S5A, conditional knockout mice with podocyte-specific ablation of CXCR4 (designated as Podo-CXCR4-/-) were generated (genotype: CXCR4^fl/fl^, Cre). Age- and sex-matched CXCR4-floxed mice (genotype: CXCR4^fl/fl^) from the same litters were used as controls (Figure S5A).

Mice with podocyte-specific deletion of CXCR4 were phenotypically normal. There was no appreciable abnormality in body weight (Figure S5B) and urinary albumin between podo-CXCR4-/- and podo-CXCR4+/+ littermates (Figure 6B). Kidney histology was normal in podo-CXCR4-/- mice (Figure S5C). Nephrin expression and distribution were intact in podo-CXCR4-/- mice (Figure 6C). In short, these data suggest that CXCR4 is dispensable for podocyte development, survival, and function *in vivo*.

### Podocyte-specific ablation of CXCR4 preserves podocyte integrity and mitigates proteinuria after injury

We next investigated the role of CXCR4 in podocyte injury by challenging podo-CXCR4-/- mice with ADR. As shown in Figure 6D, albuminuria in podo-CXCR4+/+ mice at 1 week after ADR was more severe than that in podo-CXCR4-/- mice. SDS-PAGE analysis of urine samples revealed that albumin was the major constituent of urinary proteins in mice after ADR injury (Figure 6E). Similar results were obtained at 10 days after ADR injection (Figure 6F). Of note, we presented urinary albumin in podo-CXCR4+/+ and podo-CXCR4-/- mice at 10 days after ADR, as two mice in the podo-CXCR4+/+ group died on the 12^th^ and 13^th^ day, respectively.

We examined CXCR4 expression in the kidneys of podo-CXCR4+/+ and podo-CXCR4-/- mice at 2 weeks after ADR injection. As shown in Figure 6G, immunohistochemical staining for CXCR4 revealed a podocyte-specific loss of CXCR4 protein in the kidneys of podo-CXCR4-/-mice. We further assessed podocyte injury by examining the expression of podocyte-specific markers, including WT1, nephrin and podocalyxin. As shown in Figure 6G-H, WT1-positive cells were increased in podo-CXCR4-/- glomeruli at 2 weeks after ADR, compared to podo-CXCR4+/+ controls. Western blot analyses demonstrated that nephrin and podocalyxin proteins were significantly increased in podo-CXCR4-/- mice after ADR injury (Figure 6I-J). Similar results were obtained when nephrin and podocalyxin were assessed by immunofluorescence staining (Figure 6K).

### Podocyte-specific ablation of CXCR4 blocks β-catenin signaling *in vivo*

**Figure 7:**
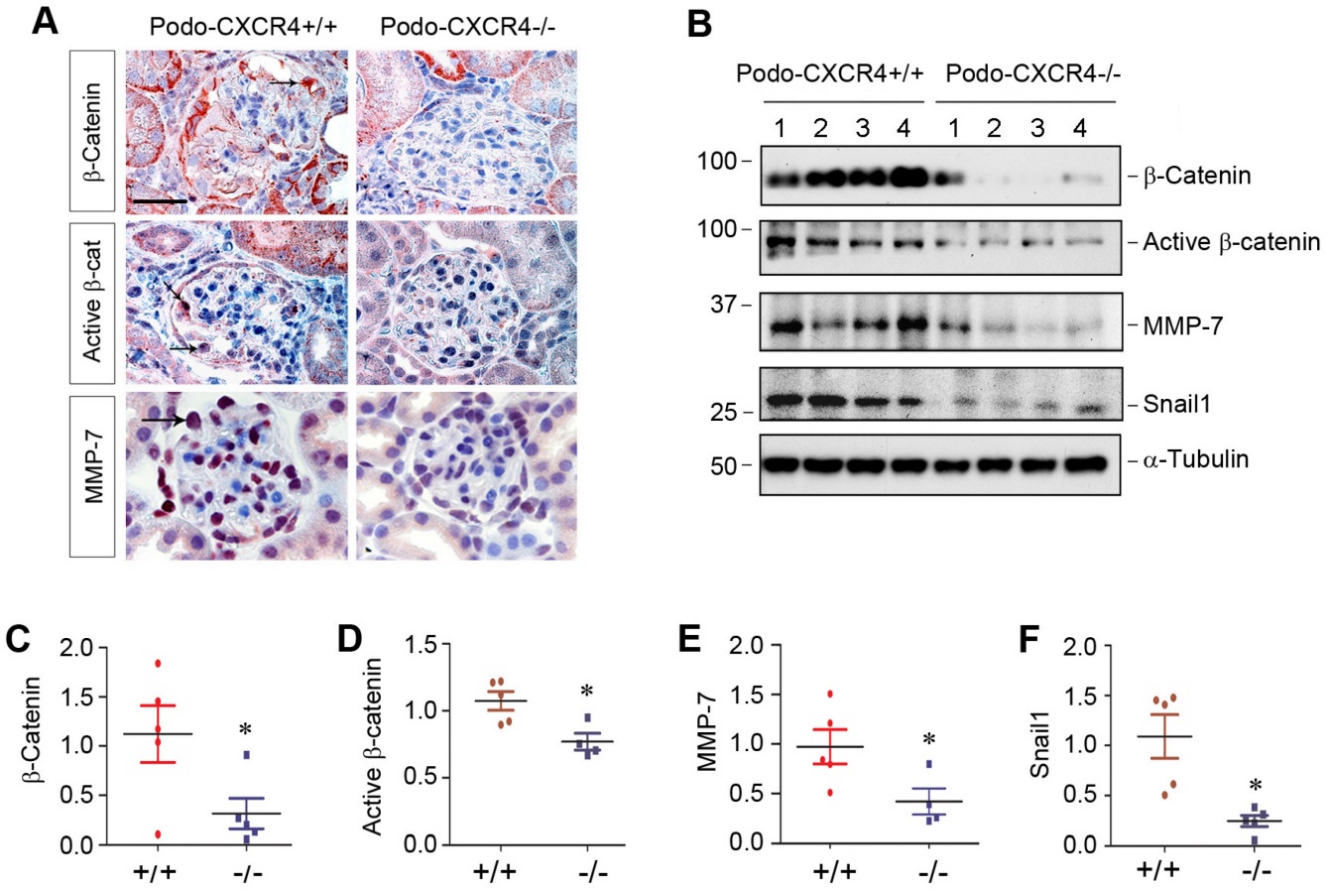
** Podocyte-specific ablation of CXCR4 inhibits β-catenin signaling in ADR model.** (**A**) Representative micrographs show the immunohistochemical staining of glomerular β-catenin, active β-catenin and MMP-7 in WT and KO mice at 2 weeks after ADR. Arrows indicate positive staining. Scale bar, 20 µm. (**B**-**F**) Western blot analyses show that podocyte-specific ablation of CXCR4 reduced renal expression of β-catenin, active β-catenin, MMP-7 and Snail1 proteins at 2 weeks after ADR. Representative Western blot (**B**) and quantitative data of β-catenin (**C**), active β-catenin (**D**), MMP-7 (**E**) and Snail1 (**F**) proteins in different groups as indicated. Numbers (1-4) indicate each individual animal in a given group. ^*^P < 0.05 versus +/+ (n = 5).

To establish the connection between podocyte injury induced by CXCR4 and β-catenin signaling *in vivo*, we examined β-catenin activation and its downstream target proteins in podo-CXCR4+/+ and podo-CXCR4-/- mice after ADR injury. Immunohistochemical staining revealed that total β-catenin, active β-catenin and its downstream MMP-7 were highly expressed in glomerular podocytes of podo-CXCR4+/+ mice at 2 weeks after ADR injection (Figure 7A). However, podocyte-specific ablation of CXCR4 largely abolished the expression of these proteins in the glomerular podocytes (Figure 7A). Similarly, Western blot analyses also show that podocyte-specific ablation of CXCR4 reduced renal expression of β-catenin, active β-catenin, MMP-7 and Snail1 at 2 weeks after ADR injection (Figure 7B-F). A reduced mRNA expression of plasminogen activator inhibitor-1 (PAI-1), another target protein of β-catenin signaling 38, in podo-CXCR4-/- mice was also observed at 2 weeks after ADR injection, compared to podo-CXCR4+/+ controls (Figure S6). These results indicate that podocyte-specific ablation of CXCR4 blocks β-catenin activation and its downstream gene expression in response to ADR injury *in vivo*.

### Podocyte-specific ablation of CXCR4 ameliorates glomerulosclerosis in ADR nephropathy

**Figure 8:**
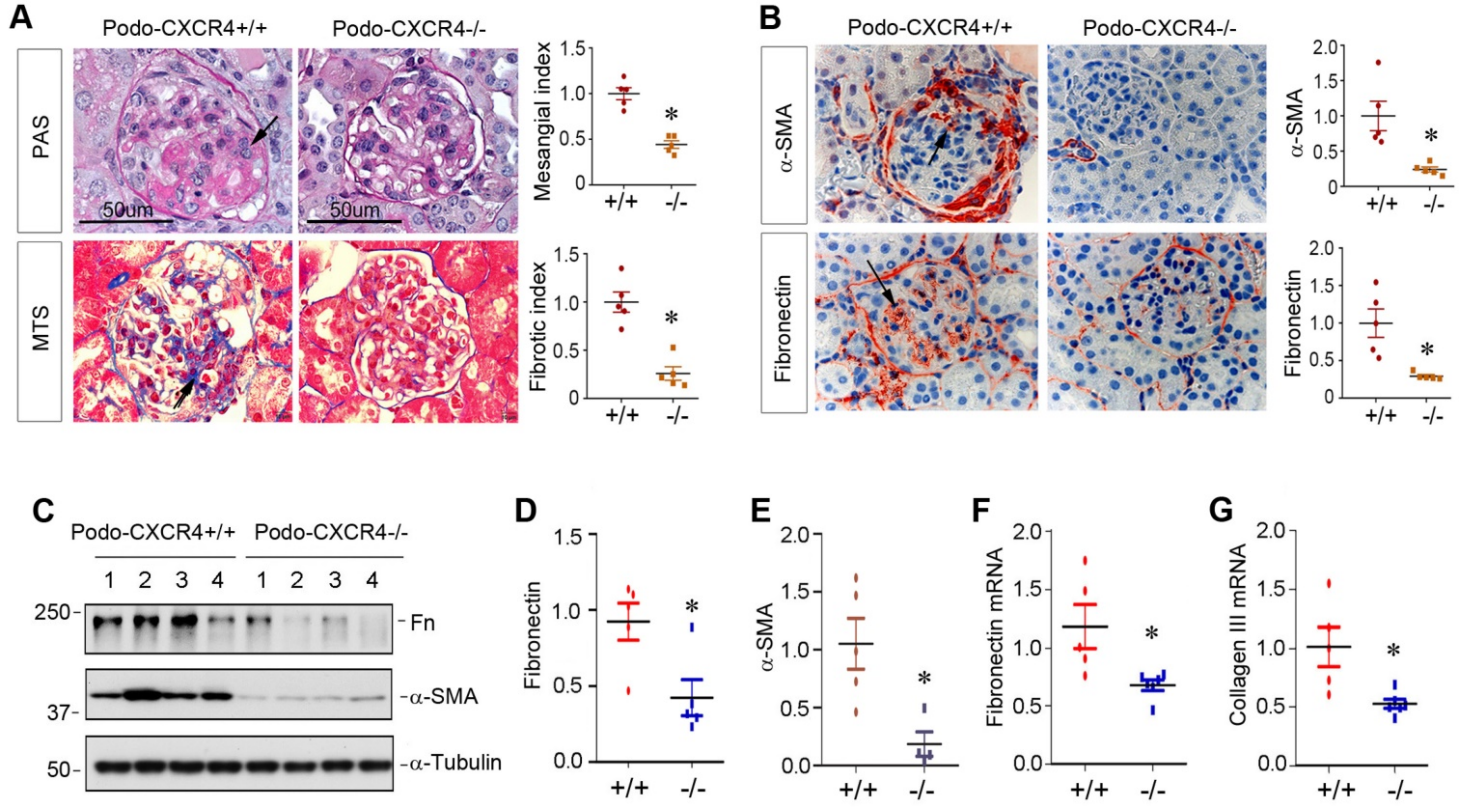
** Podocyte-specific ablation of CXCR4 ameliorates glomerulosclerotic lesions after ADR injury.** (**A**) Representative micrographs show mesangial expansion and collagen deposition by PAS and Masson's trichrome staining (MTS) in the glomeruli at 2 weeks after ADR in different groups as indicated. Arrows indicate positive staining. Scale bar, 50 µm. Relative mesangial index and fibrotic index are shown. ^*^P < 0.05 (n = 5). (**B**) Representative micrographs show α-SMA and fibronectin expression in the glomeruli at 2 weeks after ADR. Arrows indicate positive staining. Relative levels of α-SMA and fibronectin in different groups as indicated are shown. ^*^P < 0.05 (n = 5). (**C**-**E**) Western blot analyses show that podocyte-specific ablation of CXCR4 reduced renal expression of fibronectin (Fn) and α-SMA at 2 weeks after ADR. Representative Western blot (**C**) and quantitative data of fibronectin (**D**) and α-SMA (**E**) are presented. ^*^P < 0.05 (n = 4-5). (**F**, **G**) Quantitative real-time RT-PCR analyses (qRT-PCR) show a reduced mRNA expression of fibronectin (**F**) and type III collagen (**G**) in podo-CXCR4-/- mice at 2 weeks after ADR, compared to podo-CXCR4+/+ counterparts. ^*^P < 0.05 (n = 5-6).

We finally investigated the effect of podocyte-specific ablation of CXCR4 on glomerulosclerotic lesions in ADR nephropathy. As illustrated in Figure 8A, periodic acid-Schiff (PAS) staining and Masson's trichrome staining (MTS) exhibited that ADR induced mesangial expansion, collagen deposition and glomerulosclerotic lesions in podo-CXCR4+/+ mice, whereas podocyte-specific ablation of CXCR4 substantially reduced these lesions. Similarly, immunostaining for α-SMA and fibronectin also revealed that podocyte-specific ablation of CXCR4 largely abolished the expression of α-SMA and fibronectin in the glomeruli (Figure 8B). Western blot analyses further confirmed that podocyte-specific ablation of CXCR4 inhibited the expression of fibronectin and α-SMA in mouse kidneys after ADR injury (Figure 8C-E). The mRNA expression of fibronectin and collagen III was also repressed in podo-CXCR4-/- mice at 2 weeks after ADR, compared to podo-CXCR4+/+ controls (Figure 8F-G).

## Discussion

In this study, we show that activation of CXCR4 by SDF-1α *in vitro* or ADR *in vivo* recruits β-arrestin-1 and Src to form the CXCR4/β-arrestin-1/Src signalosome, which transactivates EGFR leading to subsequent phosphorylation of ERK1/2 and GSK-3β. The phosphorylation of GSK-3β leads to its inactivation, resulting in the dephosphorylation and stabilization of β-catenin (Figure 4J). This cascade of events causes β-catenin activation and its nuclear translocation, wherein it interacts with TCF/LEF transcription factors to stimulate the transcription of its target genes, resulting in podocyte injury/dysfunction and proteinuria. These studies provide significant insights into the molecular mechanism by which CXCR4 activation causes podocyte injury and proteinuria. Our findings also underscore a novel Wnt-independent pathway that leads to β-catenin activation in glomerular podocytes in the evolution of proteinuric kidney disease.

An interesting finding of the present study is that the SDF-1α/CXCR4 induces podocyte injury by activating β-catenin through a Wnt-independent pathway. Although increasing evidence indicates that Wnt/β-catenin is implicated in mediating proteinuric CKD including ADR nephropathy, most previous studies have focused on the canonical Wnt signaling 7,12. For instance, advanced oxidation protein products (AOPPs), a biomarker and mediator of reactive oxidative species (ROS), have been shown to induce Wnt ligands such as Wnt1 and Wnt7a in podocytes, which leads to β-catenin activation and podocyte dysfunction and damage 7. Studies also demonstrate that TGF-β1 promotes podocyte injury and proteinuria by inducing Wnt1 expression 39. However, CXCR4 stimulation by SDF-1α does not affect the expression of Wnt ligands at 0.5 and 24 h (Figure 2E and Figure S2), although it clearly induces β-catenin activation in podocytes (Figure 2). Notably, β-catenin activation triggered by SDF-1α/CXCR4 takes place very quickly, occurring as early as 30 min after SDF-1α stimulation, which is followed by β-catenin nuclear translocation (Figure 2). These observations suggest that SDF-1α/CXCR4 activates β-catenin by a mechanism independent of Wnt induction. This speculation is also in line with numerous earlier observations showing that a diverse array of factors, such as Hippo, YAP/TAZ, hedgehog and Smad7, can activate β-catenin in a Wnt-independent fashion 40-44. It is also reported that SDF-1α/CXCR4 signaling can activate β-catenin in colorectal and pancreatic cancer cells 20,21. In addition, degradation of E-cadherin by MMP-7 also leads to the dissociation of E-cadherin/β-catenin complex, resulting in the liberation and subsequent activation of β-catenin in kidney tubular cells 19. Activation of cannabinoid receptor type 2 (CB2) also induces the formation of a β-arrestin-1/Src/β-catenin complex in renal tubular cells, which further triggers the nuclear translocation of β-catenin and causes fibrotic response 45. Taken together, these findings underscore that Wnt-independent β-catenin activation could be a broad non-canonical mechanism that is operated in multiple cells under many circumstances.

The present study also delineates the mechanism how CXCR4 activates β-catenin in a Wnt-independent manner in podocytes. As a G protein-coupled receptor, activation of CXCR4 renders it to recruit scaffold protein β-arrestin-1, which can regulate CXCR4-mediated signaling 28,46. We show herein that CXCR4 activation by SDF-1α recruits both β-arrestin-1 and Src, a non-receptor tyrosine protein kinase, and form a large protein complex/signalosome containing CXCR4/β-arrestin-1/Src in podocytes, which leads to β-catenin activation (Figure 3). There are several studies demonstrating that β-arrestin-1 plays a role in β-catenin activation in different settings. For example, β-arrestin-1 is shown to regulate Wnt/β-catenin through a DVL-dependent pathway in HEK-293 cells 47. Activation of endothelin-A receptor by endothelin-1 also activates β-catenin by β-arrestin-1-mediated transactivation of EGFR 33. In the present study, we have shown that depletion of β-arrestin-1 abolishes the activation of β-catenin induced by SDF-1α/CXCR4 (Figure 3). These results corroborate a crucial role of β-arrestin-1 in mediating CXCR4-induced β-catenin activation in podocytes.

This study has also uncovered how the formation of CXCR4/β-arrestin-1/Src signalosome leads to β-catenin activation. It appears that the formation of CXCR4/β-arrestin-1/Src signalosome triggers a sequential phosphorylation of Src, EGFR, ERK1/2 and GSK-3β (Figure 4). This conclusion is supported by the dynamics and sequences of the phosphorylation of these proteins, as the abundances of p-Src and p-EGFR are increased as early as 5 min after incubation with SDF-1α in podocytes, while p-ERK1/2 and p-GSK-3β levels start to increase at 15 min and reach to the peak at 30 min, respectively (Figure 4). The phosphorylation of ERK1/2 and GSK-3β is blocked by either knocking down of β-arrestin-1 or treatment with U0126, the highly selective inhibitor of ERK upstream kinases MEK1 and MEK2. This cascade of phosphorylation of signal proteins is also in line with many previous reports 34,35. For instance, ERK activation has been shown to cause GSK-3β phosphorylation and inactivation, leading to β-catenin activation 48. Taken together, it is conceivable that CXCR4 activation in podocytes recruits β-arrestin-1 and Src, leading to the formation of CXCR4/β-arrestin-1/Src signalosome, which causes Src phosphorylation and leads to transactivation of EGFR and its downstream ERK1/2, thereby triggering GSK-3β inactivation and β-catenin stabilization and activation (Figure 4J). Consistent with this notion, inhibition of β-catenin signaling by ICG-001, a small molecule inhibitor that blocks β-catenin-mediated gene transcription 26, preserves podocyte integrity and prevents against podocyte damage by SDF-1α in glomerular mini-organ culture *ex vivo* (Figure 5).

The important role of CXCR4 in podocyte injury and proteinuria is unambiguously confirmed in mice with podocyte-specific ablation of CXCR4 *in vivo*. Consistent with podocyte-specific deletion of β-catenin 12, ablation of CXCR4 does not cause overt abnormality in mice under normal physiological conditions, suggesting that CXCR4 is dispensable for podocyte maturation, survival and function. This is not totally surprising, as the levels of CXCR4 and β-catenin in normal glomeruli are hardly detectable (Figure 1). However, podocyte-specific deletion of CXCR4 markedly reduces proteinuria after challenged with ADR and preserves podocyte integrity by restoring the expression of WT1, nephrin and podocalyxin (Figure 6). These beneficial effects are associated with the inhibition of β-catenin, accompanied by repression of β-catenin target genes such as MMP-7, Snail1 and PAI-1 after ADR challenge (Figure 7). These observations underline the pathogenic action of CXCR4 in glomerular podocytes by activating β-catenin signaling. Therefore, targeted inhibition of this signal pathway might be an effective strategy for the prevention and treatment of proteinuric CKD.

The present study has some limitations, in which only ADR model of podocyte injury and proteinuria was used. In view of that CXCR4 and β-catenin are upregulated in the glomeruli of several CKD models (Figure 1), future studies using other models of glomerular diseases or intervention strategies are warranted. Furthermore, because mice with C57BL/6J background are generally resistant to ADR injury, high dose of this drug is required to trigger podocyte injury and proteinuria, which leads to animal loss and reduces the sample size. Of interest, the loss of animals only occurs in the podo-CXCR4+/+ mice within 2 weeks, but not in the podoc-CXCR4-/- counterparts, implying that the renoprotective role of podocyte-specific deletion of CXCR4 could be underestimated in this study. More studies are needed in this area.

In summary, we show herein that CXCR4 mediates podocyte injury and proteinuria by activating β-catenin via a cascade of events involving β-arrestin-1, Src, EGFR, ERK1/2, GSK-3β. Podocyte-specific deletion of CXCR4 blunts β-catenin activation, ameliorates podocyte injury, reduces proteinuria and glomerulosclerosis, thereby halting the progression of CKD. This study provides significant insights into the mechanism by which CXCR4 mediates podocyte injury. Targeting any key step in the cascade of this signaling may open new avenues for developing the treatment of proteinuric kidney diseases.

## Supplementary Material

Supplementary figures and tables.
