# Supplementary material for: CXCR4 induces podocyte injury and proteinuria by activating β-catenin signaling
Source: Theranostics. 2022 Jan 1;12(2):767–81. doi: 10.7150/thno.65948 (PMC8692909; doi:10.7150/thno.65948)
Supplement: Supplementary file 1 — Supplementary figures and tables. [file thnov12p0767s1.pdf]

CXCR4 induces podocyte injury and proteinuria by activating β-catenin signaling

Hongyan Mo,[1,2,3] Qian Ren,[1] Dongyan Song,[1] Bo Xu,[1] Dong Zhou,[2,4] Xue Hong,[1] Fan Fan Hou,[1] Lili Zhou,[1]·Youhua Liu[1,2]

Supplementary Figures

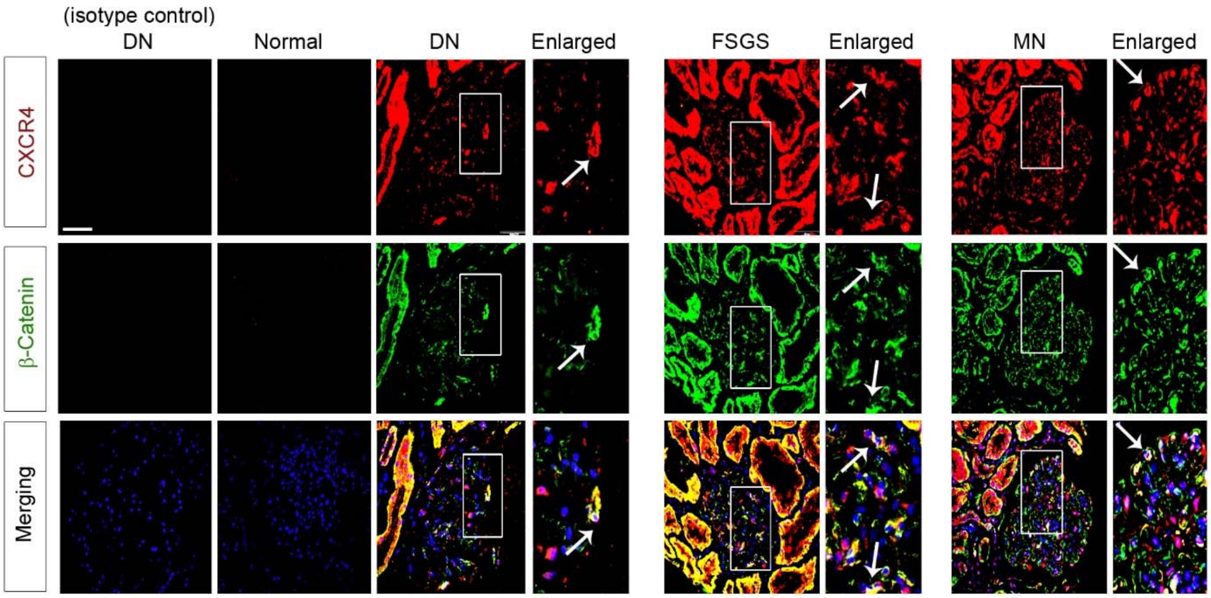

Figure S1. Colocalization of CXCR4 and β-catenin in the glomeruli of kidney biopsies from patients with proteinuric kidney diseases. Frozen sections were immunostained for CXCR4 (red) and β-catenin (green), respectively. Colocalizations of CXCR4 and β-catenin in glomeruli are indicated by arrows. Scale bar, 50 μm. Representative micrographs of normal kidney (1 case), DN (2 cases), FSGS (2 cases) and MN (2 cases). DN, diabetic nephropathy; FSGS, focal and segmental glomerulosclerosis; MN, membranous nephropathy.

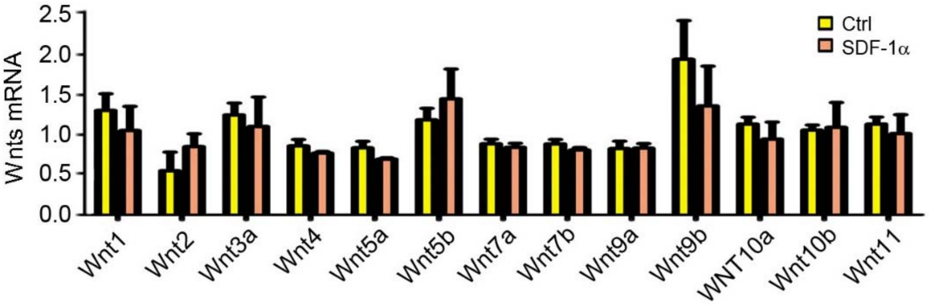

Figure S2. SDF-1α does not affect the mRNA expression of various Wnt ligands in cultured podocytes. Quantitative real-time PCR (qPCR) analyses show mRNA expression of Wnt ligands in cultured podocytes (MPC5) after treatment with SDF-1α (100 ng/mL) for 24 h (n = 3).

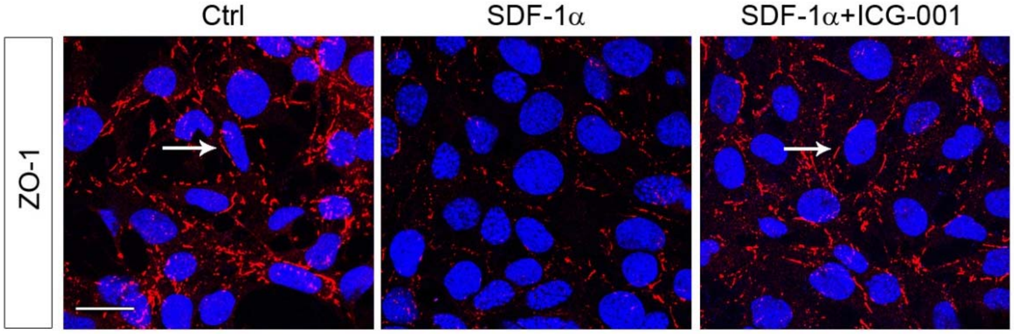

Figure S3. Blockade of β-catenin signaling by ICG-001 preserves tight junction protein ZO-1 in cultured podocytes. Representative micrographs show ZO-1 staining in the cell-cell junctions of podocytes. Arrow indicates positive staining for ZO-1. Scale bar, 15 µm.

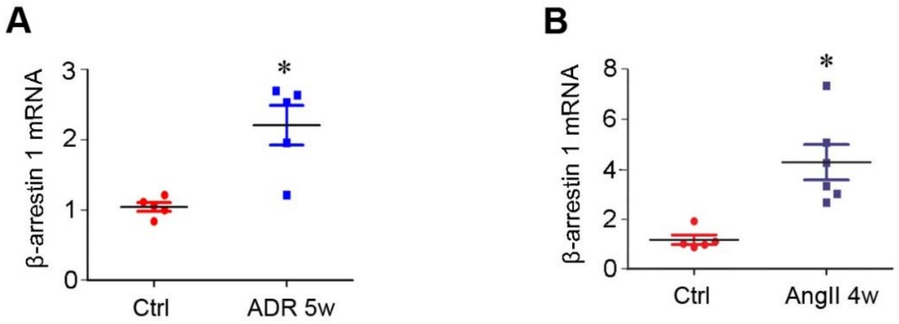

Figure S4. β-arrestin-1 is upregulated in various models of CKD. (**A**) Graphical representation shows the relative mRNA levels of β-arrestin-1 in control or ADR for 5 weeks as indicated. $^{*}P < 0.05$ versus controls (n = 5). (**B**) Graphical representation shows the relative mRNA levels of β-arrestin-1 in control or angiotensin II infusion for 4 weeks as indicated. $^{*}P < 0.05$ versus controls (n = 5-6).

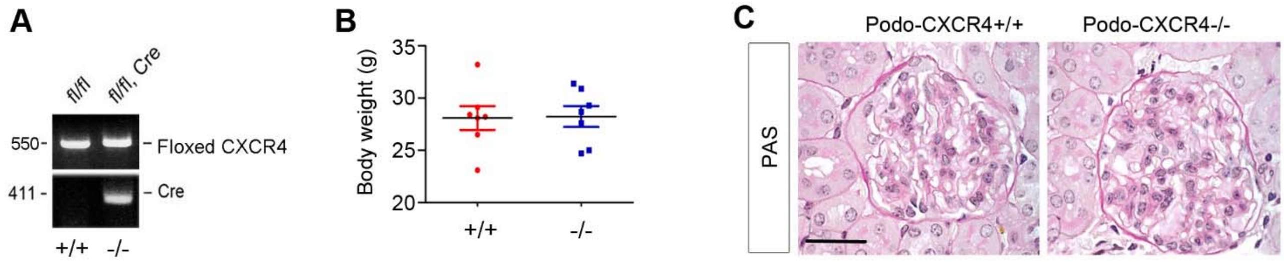

Figure S5. Generation of conditional knockout mice with podocyte-specific ablation of CXCR4. (**A**) Genotyping of the mice by PCR analysis of genomic DNA. Lane 1 shows the genotyping of the control mice used in this study (genotype: $CXCR4^{fl/fl}$), whereas lane 2 denotes the genotyping of the podocyte-specific CXCR4 knockout mice (genotype: $CXCR4^{fl/fl}$ Cre), designated as podo-CXCR4 -/-. (**B**) Body weights of podo-CXCR4+/+ and podo-CXCR4-/- mice at 3 months of age. P=0.927 (n = 7). (**C**) Representative micrographs show PAS staining in podo-CXCR4+/+ and podo-CXCR4-/- mice in normal conditions. Scale bar, 20 μm.

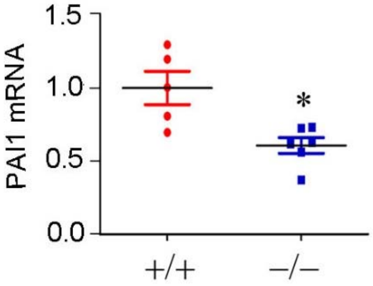

Figure S6. Ablation of CXCR4 in podocytes reduces PAI-1 expression after ADR injury. Quantitative real-time RT-PCR (qRT-PCR) analyses show a reduced mRNA expression of PAI-1 in podo-CXCR4-/- mice at 2 weeks after ADR, compared to podo-CXCR4+/+ mice. $^{*}P < 0.05$ (n = 5-6).

Table S1. Nucleotide sequences of the primers used for qPCR

Mouse gene	Primer Sequence 5' to 3'	
	Forward	Reverse
Cre recombinase	AGGTGTAGAGAAGGCACTTAGC	CTAATCGCCATCT TCCAGCAGG
CXCR4	CCACCCAGGACAGTGTGACTCTAA	GATGGGATTTCTGTATGAGGATTAGC
Wnt1	CGACTGATCCGACAGAACCC	AGCCTCGGTTGACGATCTTG
Wnt2	GCTCTTGACCTGGCTCACCC	CAGGCCAATGGCACGCAT
Wnt2b	GGAATGGATCCGAGAGTGCC	GATAGCGTGGACCACTCCTG
Wnt3	CCTCGCTGGCTACCCAATTT	GCTGGGCATGATCTCGATGT
Wnt3a	GAGGCCACGTTACACGTACC	ACCCATCTATGCCATGCGAG
Wnt4	ATCTCTICAGCAGGTGTGGC	TGTTGTCCGAGCATCCTGAC
Wnt5a	AGACCTCAGAGGGGATGGAC	TCTCCGTGCACTTCTTGCAT
Wnt5b	GTGCAGAGACCGGAGATGTT	AGCTGTCTCTCGGATGTCCT
Wnt6	TTCGGGGATGAGAAGTCAAG	CGGCACAGACAGTTCTCCTC
Wnt7a	CGGACGCCATCATCGTCATA	CACTTTGAGCTCCTTCCCGA
Wnt7b	TCCTCTACGTGAAGCTCGGA	TCCCCGATCACAATGATGGC
Wnt8a	GCCTATCTGACCTACACCGC	GGATGTCTCTCTCGTGGCAG
Wnt8b	ACTAGAAACTGCAGCCTCGG	CTCGAGGGCATCCACAAACT
Wnt9a	AACCTCGTGGGTGTGAAGGT	TTGTGTTTTAGGTGCTTGCCC
Wnt9b	CAGGTGCTGAAGCTACGCTA	CTTCCATGTAGACCAGGTCCC
Wnt10a	GGTAAACTGAAGGCTTGCGG	AAGTATGGCCGGGTGTTCAG
Wnt10b	CAAGACCGGCTTAGAGCCAA	TCCATGTCGTGGTTACAGCC
Wnt11	ACTGTAAACAGCTGGAGGGC	CGATGGAGGAGCAGTTCCAG
Wnt16	TACGGCATGTGGTTCAGCAG	GCGGCAGTCCACAGACATTA
Fibronectin	CGAGGTGACAGAGACCACAA	CTGGAGTCAAGCCAGACACA
COL3A1	AGGCAACAGTGGTTCTCCTG	GACCTCGTGCTCCAGTTAGC
PAI-1	TGGAAAGAGCCAGATTTATCAT	GAAGTAGAGGGCATTCACCAG
β-arrestin1	GGCGACAAAGGGACACGAG	GTGTCACGTAGACTCGCCTT
β-actin	CAGCTGAGAGGGAAATCGTG	CGTTGCCAATAGTGATGACC

Table S2. The sources of antibodies used in this study

Antibodies	Catalogue number	Company	Location
**Primary antibodies**			
anti-CXCR4	sc-6190	Santa Cruz Biotechnology	Santa Cruz, CA
anti-CXCR4	sc-53534	Santa Cruz Biotechnology	Santa Cruz, CA
anti-CXCR4	ab2074	Abcam	Cambridge, MA
anti-CXCR4	PA5-19856	Fisher Scientific	Pittsburgh, PA
anti-SDF-1α	A00053-2	Boster Biological Technology	Wuhan, China
anti-α-SMA	A2547	Sigma-Aldrich	St. Louis, MO
anti-α-SMA	ab5694	Abcam	Cambridge, MA
anti-active β-catenin	05-665	EMD Millipore	Billerica, MA
anti-active β-catenin	#19807	Cell Signaling Technology	Danvers, MA
anti-β-catenin	ab15180	Abcam	Cambridge, MA
anti-β-catenin	#610154	BD Transduction Laboratories	San Jose, CA
anti-MMP7	GTX32725	GeneTex	Irvine, CA
anti-MMP7	GTX104658	GeneTex	Irvine, CA
anti-nephrin	20R-NP002	Fitzgerald Industries International	Concord, MA
anti-WT1	sc-192	Santa Cruz Biotechnology	Santa Cruz, CA
anti-podocalyxin	AF1556	R&D Systems	Minneapolis, MN
anti-fibronectin	F3648	Sigma-Aldrich	St. Louis, MO
anti-ZO-1	61–7300	Thermo Fisher Scientific	Waltham, MA
anti-β-arrestin1	12673s	Cell Signaling Technology	Danvers, MA
anti-Snail1	ab82846	Abcam	Cambridge, MA
anti-phospho-EGFR	sc-57542	Santa Cruz Biotechnology	Santa Cruz, CA
anti-EGFR	sc-373746	Santa Cruz Biotechnology	Santa Cruz, CA
anti-phospho-Src (Y418)	ab40660	Abcam	Cambridge, MA
anti-Src	ab47405	Abcam	Cambridge, MA
anti-phospho-GSK3β (Ser9)	9336s	Cell Signaling Technology	Danvers, MA
anti-GSK3β	9315s	Cell Signaling Technology	Danvers, MA
anti-phospho-p44/42MAPK(ERK1/2) (Thr202/Tyr204)	4370s	Cell Signaling Technology	Danvers, MA
anti-p44/42MAPK (ERK1/2)	9102s	Cell Signaling Technology	Danvers, MA
anti-p44/42MAPK (ERK1/2)	4695s	Cell Signaling Technology	Danvers, MA
anti-α-tubulin	T9026	Sigma-Aldrich	St. Louis, MO
anti-GAPDH	RM2002	Ray Antibody Biotech	Peachtree Corners, GA

**Secondary antibodies**			
Goat anti-mouse	BA1050	Boster Biological Technology	Wuhan, China
Goat anti-rabbit	BA1054	Boster Biological Technology	Wuhan, China
Rabbit anti-goat	BA1060	Boster Biological Technology	Wuhan, China
Goat anti-Guinea pig	106-001-003	Sigma-Aldrich	St. Louis, MO
Biotin-SP AffiniPure Goat Anti-Mouse lgG(H+L)	115-065-146	Jackson Immuno Research Laboratories	West Grove, PA
Biotin-SP AffiniPure Goat Anti-Rabbit IgG(H+L)	111-065-144	Jackson Immuno Research Laboratories	West Grove, PA
Cy3-AffiniPure Goat Anti-Guinea Pig IgG(H+L)	106-165-003	Jackson Immuno Research Laboratories	West Grove, PA
Cy3-AffiniPureRabbit Anti-Goat IgG(H+L)	305-165-003	Jackson Immuno Research Laboratories	West Grove, PA
Cy2-AffiniPure Goat Anti-Rabbit IgG(H+L)	111-225-144	Jackson Immuno Research Laboratories	West Grove, PA
Cy3-AffiniPure Goat Anti-Rabbit IgG(H+L)	111-165-003	Jackson Immuno Research Laboratories	West Grove, PA
Cy2-AffiniPure Donkey Anti-Rabbit IgG(H+L)	711-225-152	Jackson Immuno Research Laboratories	West Grove, PA
Cy3-AffiniPure Donkey Anti-Mouse IgG(H+L)	715-165-150	Jackson Immuno Research Laboratories	West Grove, PA
